# Herbal smoking products: A neglected frontier in global tobacco control

**DOI:** 10.18332/tid/216707

**Published:** 2026-02-14

**Authors:** Komal Rewatkar, Noopur Kokane, Sachin Khatri

**Affiliations:** 1 Department of Public Health Dentistry, Government Dental College and Hospital Nagpur, Nagpur, India

**Keywords:** COTPA, tobacco regulation, herbal smoking

**Dear Editor,**

During our participation in the World Conference on Tobacco Control (WCTC) in Dublin, we were inspired by the diversity of evidence-based strategies aimed at curbing tobacco use globally. However, amid discourse on electronic nicotine delivery systems (ENDS), heated tobacco products (HTPs), and combustible tobacco, one glaring omission was the issue of herbal smoking products (HSPs), an under-regulated category that is quietly gaining popularity, particularly in countries like India.

Contrary to public perception, herbal smoking products such as herbal hookahs, herbal bidis, and non-tobacco cigarettes are not harmless alternatives^1^. Although these products are marketed as nicotine-free, multiple studies have shown that their combustion generates harmful substances, including polycyclic aromatic hydrocarbons (PAHs), volatile organic compounds (VOCs), and carbon monoxide^2^. The World Health Organization (WHO) clearly states that the absence of nicotine does not eliminate the danger posed by other toxicants created during burning^3^.

In India, herbal hookah parlors have emerged as fashionable social spots, especially among adolescents and young adults^4^. Their operators exploit loopholes in national legislation such as the Cigarettes and Other Tobacco Products Act (COTPA) 2003, which currently omits herbal smoking products from the definitions governing advertising restrictions, health warnings, and public place usage^5^. This regulatory void emboldens aggressive marketing, with over 87% of online videos and 67% of Instagram content portraying herbal hookahs as ‘healthy’ and free from health risks, despite lacking credible scientific validation^4^.

HSPs may also serve as initiation products that normalize the act of smoking. This behavioral priming may pave the way for subsequent use of nicotine-containing substances, aligning with the ‘gateway hypothesis’. What makes this particularly concerning is that young users often perceive herbal products as benign due to their ‘natural’ labeling, a deceptive marketing tactic similar to those historically used by the tobacco industry to downplay harms from light or filtered cigarettes^6^.

The mirage of safety created by these products mirrors the broader harm reduction narrative advanced by the global tobacco industry in the context of e-cigarettes and HTPs. Studies have shown that such strategies, while promoted as scientifically sound, are frequently designed to maintain consumer dependence and delay cessation^7^. By promoting a false dichotomy between ‘safe’ and ‘unsafe’ forms of smoking, this narrative serves more as a profit-driven distraction than a true public health intervention^8^.

It is imperative that global and national regulatory frameworks be urgently updated to include herbal smoking products. COTPA Sections 4, 5, and 7 pertaining to public smoking bans, advertising restrictions, and pictorial health warnings should be expanded to cover all forms of combustible products, irrespective of their nicotine or tobacco content^9^. International guidelines, including the WHO Framework Convention on Tobacco Control (FCTC), can further regulate these products with enhanced scrutiny.

Public health campaigns should be recalibrated to address the risks of herbal smoking, emphasizing that ‘herbal’ does not mean harmless. Research-based awareness campaigns, school-level interventions, and social media regulation are critical to disrupting the misinformation cycle in order to safeguard the youth.

As public health professionals, we emphasize the urgent need to re-evaluate and regulate herbal smoking products. Unchecked proliferation of these products may facilitate a resurgence of smoking behaviors that circumvent existing tobacco control measures.

## Data Availability

Data sharing is not applicable to this article as no new data were created.
